# Leptin Elicits *In Vivo* Eosinophil Migration and Activation: Key Role of Mast Cell-Derived PGD_2_

**DOI:** 10.3389/fendo.2020.572113

**Published:** 2020-09-29

**Authors:** Natália R. T. Amorim, Glaucia Souza-Almeida, Tatiana Luna-Gomes, Patricia T. Bozza, Claudio Canetti, Bruno L. Diaz, Clarissa M. Maya-Monteiro, Christianne Bandeira-Melo

**Affiliations:** ^1^ Laboratório de Inflamação, Instituto de Biofísica Carlos Chagas Filho, Universidade Federal do Rio de Janeiro, Rio de Janeiro, Brazil; ^2^ Laboratório de Imunofarmacologia, Instituto Oswaldo Cruz - IOC, FIOCRUZ, Rio de Janeiro, Brazil; ^3^ Laboratório de Imunoinflamação, Instituto de Biologia, Universidade de Campinas, Campinas, Brazil; ^4^ Departamento de Ciências da Natureza, Instituto de Aplicação Fernando Rodrigues da Silveira, Universidade do Estado do Rio de Janeiro, Rio de Janeiro, Brazil

**Keywords:** eosinophils, mast cells, lipid droplets, leptin, prostaglandin D2, leukotriene C4, RANTES, TNF-alpha

## Abstract

Eosinophils are key regulators of adipose tissue homeostasis, thus characterization of adipose tissue-related molecular factors capable of regulating eosinophil activity is of great interest. Leptin is known to directly activate eosinophils *in vitro*, but leptin ability of inducing *in vivo* eosinophilic inflammatory response remains elusive. Here, we show that leptin elicits eosinophil influx as well as its activation, characterized by increased lipid body biogenesis and LTC_4_ synthesis. Such leptin-triggered eosinophilic inflammatory response was shown to be dependent on activation of the mTOR signaling pathway, since it was (i) inhibited by rapamycin pre-treatment and (ii) reduced in PI3K-deficient mice. Local infiltration of activated eosinophils within leptin-driven inflammatory site was preceded by increased levels of classical mast cell-derived molecules, including TNFα, CCL5 (RANTES), and PGD_2_. Thus, mice were pre-treated with a mast cell degranulating agent compound 48/80 which was capable to impair leptin-induced PGD_2_ release, as well as eosinophil recruitment and activation. In agreement with an indirect mast cell-driven phenomenon, eosinophil accumulation induced by leptin was abolished in TNFR-1 deficient and also in HQL-79–pretreated mice, but not in mice pretreated with neutralizing antibodies against CCL5, indicating that both typical mast cell-driven signals TNFα and PGD_2_, but not CCL5, contribute to leptin-induced eosinophil influx. Distinctly, leptin-induced eosinophil lipid body (lipid droplet) assembly and LTC_4_ synthesis appears to depend on both PGD_2_ and CCL5, since both HQL-79 and anti-CCL5 treatments were able to inhibit these eosinophil activation markers. Altogether, our data show that leptin triggers eosinophilic inflammation *in vivo via* an indirect mechanism dependent on activation of resident mast cell secretory activity and mediation by TNFα, CCL5, and specially PGD_2_.

## Introduction

Eosinophils are recognized as classical effectors of Type 2 immune responses; and yet, eosinophil anti-helminthic and allergy-related deleterious roles are continually reexamined, with frequent descriptions of novel eosinophil-driven molecular mechanisms emerging all the time (1). Moreover, recent groundbreaking studies had further widened the understanding of eosinophil functions from disease-related pro-inflammatory to homeostatic immunomodulatory roles (2, 3). Eosinophils are now acknowledged as key regulators of adipose tissue homeostasis, with roles in control of thermogenic energy expenditure and resident macrophages modulation (4, 5). Therefore, characterization of adipose tissue-related molecular factors capable of regulating eosinophil activity is of particular interest.

Leptin is a pleiotropic adipose tissue-derived cytokine with significant immuno-neuro-endocrine roles (6). Leptin is considered a key factor to integrate adipose tissue with the systemic metabolism (7). Also, many studies have demonstrated important roles of leptin within the adaptative and innate immune systems regulation, inflammation and response to infection (8–12). Our hypothesis is that leptin is a key physiological stimulus that modulates adipose eosinophils, since (i) significant leptin levels are present in either lean or obese adipose tissue; (ii) adipose eosinophils are continuously exposed to adipocyte-derived leptin; (iii) the full length leptin receptor is present on eosinophil surface (13); and (iv) leptin is known to activate human and mouse eosinophils *in vitro*. Indeed, stimulation of eosinophils with leptin directly elicits a variety of activities, including: survival, chemokinesis (14, 15), secretion of cytokines (14), lipid body biogenesis, as well as synthesis of lipid mediators as prostaglandins (PGs) and leukotrienes (16). Straightforward research aiming to characterize the direct impact of *in situ* leptin on triggering eosinophil recruitment and activation are still missing, despite the fact that some reports indicate that leptin may regulate eosinophils in low- *versus* high-fat diets mouse models of obesity (17, 18).

Numerous studies on the cellular mechanisms governing allergy-driven inflammatory diseases have established mast cells as canonical orchestrators of eosinophilic inflammation (19, 20). Ubiquitously distributed in tissues and preferentially localized in close proximity to vascular vessels, mast cells are strategically localized and ready to coordinate eosinophil recruitment and activation under specific stimulation. Among mast cell repertoire of molecules responsible for eliciting both eosinophil migration to sites of allergic reaction and *in situ* activation of infiltrating eosinophils, important examples are the cytokines IL-5 and eotaxin (19, 20), as well as *de novo* synthesized lipid mediators notably PGD_2_ (21). Furthermore, mast cells do express bioactive leptin receptors (22), whose stimulation is able to trigger activation of mast cells secretory activities (23). More importantly, mast cells are able to intermediate leptin effects in even non-conventional mast cell-regulated physiological conditions, like for instance leptin-induced altered sympathetic activity (24), diarrhea-predominant irritable bowel syndrome (25) or coronary atherosclerosis (26). Therefore, it seems reasonable to hypothesize that activation of leptin receptor in mast cells would be capable to elicit one of the utmost archetypal functions of mast cells—the induction of eosinophilic reactions.

Our study aims to investigate the ability of leptin to trigger *in vivo* eosinophilic inflammation, characterized by cell migration and cellular activation. Without any disregard to potential direct impacts of exogenous leptin on eosinophils *in vivo*—as well-established by a series of *in vitro* studies, including one of ours (16)—here, efforts were focused on characterizing indirect mast cell-driven effects of leptin on triggering eosinophilic response *in vivo*. We further defined the roles of mast cell-derived molecules on leptin-elicited eosinophil response, identifying PGD_2_ as a key mediator.

## Material and Methods

### Animals

We used male mice of different strains: C57BL/6, BALB/cBALB/c tumor necrosis factor receptor 1-deficient (TNFR1^−/−^; C57BL/6 background), and PI3Kγ-deficient (PI3Kγ^−/−^; C57BL/6 background) mice and respective wild types (WTs), obtained as previously described (27). Mice were from either the CCS/UFRJ or FIOCRUZ breeding centers, raised and maintained under the same housing conditions. All animal care and experimental protocols were conducted following the guidelines of the Brazilian Council for Care and Use of Experimentation Animals (CONCEA). The Animal Use Welfare Committees of both Federal University of Rio de Janeiro (CEUA-CCS/UFRJ license number 090/18) and Oswaldo Cruz Institute (CEUA-IOC license number L-011/2015) approved all protocols used in this study.

### Mouse Models of Leptin-Induced Eosinophilic Inflammation

Two distinct approaches of leptin *in vivo* stimulation to trigger eosinophil inflammatory response were performed: (i) intraperitoneal (i.p.) administration of murine leptin (0.5, 1, or 2 mg/kg; Peprotech) or its vehicle (sterile LPS-free saline) in naive mice of C57BL/6 background (as indicated) (8); or (ii) intrapleural (i.pl.) injection of leptin (0.5, 1, or 2 mg/kg) or its vehicle (sterile LPS-free saline) in naïve or in previously sensitized BALB/cBALB/c mice (as indicated). As previously described (28), mice were sensitized with a subcutaneous (s.c.) injection (0.2 ml) of ovalbumin (OVA; 50 µg; Sigma-Aldrich) and Al(OH)_3_ (5 mg) in 0.9% NaCl solution (sterile saline) at days 1 and 7. Animals were euthanized 6 or 24 h after leptin administration. Peritoneal and pleural cavities were rinsed with 3 and 0.5 ml of HBSS (Hank’s balanced salted solution), respectively. Total leukocyte count was performed in Neubauer chambers and differential eosinophil count in May-Grunwald-Giemsa stained cytospin slides in a blinded fashion.

Treatments to study the involvement of mTOR pathway in leptin-elicited eosinophil influx, C57BL/6 mice received three i.p. injections of rapamycin (12.5 μg/kg; Sigma-Aldrich), 12 h and 15 min before, and 12 h after leptin or saline challenge (8), followed by the peritoneal lavage at 24 h after challenge. For determination of CCL5 and PGD_2_ potential role in leptin-induced eosinophilic inflammation, sensitized BALB/cBALB/c mice were treated, respectively, either by means of i.pl. injection of neutralizing antibody anti-CCL5 (10 µg/cavity; Peprotech) or i.p. injection of selective inhibitor of PGD synthase HQL-79 (1 mg/kg; Cayman Chemicals), both 1 h before leptin injection. To investigate the participation of resident mast cells on leptin-induced eosinophilic reaction, sensitized BALB/cBALB/c mice were treated by means of local (i.pl.) injection of mast cell degranulating agent compound 48/80 (12 µg/cavity; Sigma-Aldrich) 72 h before leptin i.pl. administration (29). The efficacy of a selective impact on resident mast cell population was ascertained by absence of toluidine blue-stained cells in pleural lavage with no changes in other pleural cell populations.

### Production and Stimulation of Bone Marrow-Derived Mast Cells (BMMC)

With slight modifications, mast cells were differentiated *in vitro* from mouse bone marrow cells as previously described (30). Briefly, femurs and tibiae bone marrow of BALB/cBALB/c mice were rinsed with RPMI 1640, and cells were then cultured at 10^3^ cells/ml in medium containing IL-3 (2 ng/ml; Peprotech), 20% FBS (VitroCell), 100 IU/ml penicillin, and 10 μg/ml streptomycin (cell medium was replaced on days 7, 14, 21, and 28). After 4 weeks, more than 99% of the cells in the culture were mast cells with characteristic metachromatic granules as assessed by toluidine blue staining. Cell viability was always >95% as determined by Trypan blue exclusion.

To study mast cell activation, bone marrow-differentiated mast cells (3 × 10^6^ cells/ml) in HBSS were pre-treated or not with HQL-79 (10 µM) for 30 min and then incubated with murine leptin (50 nM; Peprotech) for 1 h at 37°C. Cells were then pelleted at 200 × *g*, and supernatants were stored at −80°C for further measurements. It is noteworthy to mention that classical mast cell stimuli, including IgE receptor crosslinking or SCF, typically evoke at least 100× larger responses than leptin effect, reaching levels of ng of PGD_2_ or LTC_4_/10^6^ BMMC within 1 h of *in vitro* stimulation.

### Measurements of Mediators Release

The lipid mediators PGD_2_ and cysteinyl LTs (cysLTs; as a surrogate marker of LTC_4_ synthesis) found either in cell-free pleural fluids or mast cell supernatants were detected by commercial EIA kits, according to the manufacturer´s instructions (Cayman Chemicals). The levels of TNFα and CCL5 found in cell-free peritoneal lavages or pleural fluids were quantified by Duo Set ELISA kits, according to manufacturer’s protocol (R&D Systems). For determination of secretory granule exocytosis, release of β-hexosaminidase was measured in the supernatant and lysed cell pellets, as described (31, 32).

### Lipid Body Staining and Enumeration

For lipid body counting, cells in cytospin slides were fixed in 3.7% formaldehyde and stained with 1.5% OsO_4_ in 0.1 M cacodylate buffer, as previously described (16). Fifty consecutively eosinophils/slide were evaluated in a blinded fashion by more than one observer by bright field microscopy.

### Statistical Analysis

Results are expressed as the mean ± SEM. *In vivo* data were analyzed by one-way ANOVA followed by Student-Newman-Keuls test. *In vitro* results regarding leptin-stimulated mast cells were analyzed by paired *t*-test. Differences were considered to be significant when *p* < 0.05.

## Results

### Leptin Elicits Both Eosinophil Migration and Activation *In Vivo*

**Figure 1:**
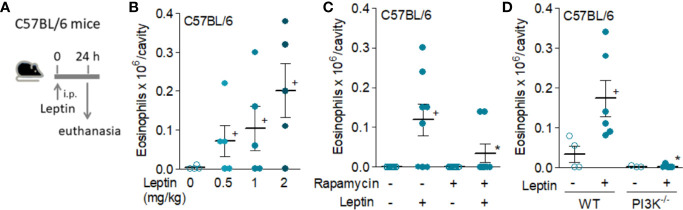
*Leptin induces in vivo eosinophil migration in naive C57/BL6 mice by a mechanism involving activation of mTOR and PI3K.* Panel **(A)** shows a brief schematic representation of the peritonitis model induced by i.p. injection of leptin in C57BL/6 mice employed in this study [data shown in **(B–D)**]. Leptin was injected into peritoneal cavities of naïve C57BL/6 mice. Peritoneal cells were collected and stained by May–Grünwald–Giemsa for eosinophil recruitment analysis within 24 h of leptin stimulation. In **(B)**, different concentrations of leptin (0.5, 1, and 2 mg/kg) were used. In **(C)**, pre-treatment with rapamycin (12.5 μg/kg per injection) 12 h before, 15 min before, and 12 h after leptin injection (1 mg/kg) was performed. In **(D)**, naïve wild type or PI3K deficient C57BL/6mice received i.p. injection of leptin (1 mg/kg) and peritoneal fluids were collected within 24 h of stimulation. There are no significant differences between the number of peritoneal eosinophils recovered from the control groups of wild type and PI3Kγ−/− C57BL/6 mice. Values are expressed as the mean ± SEM (experiments were repeated at least once). ^+^ *p* < 0.05 compared to control group.*p < 0.05 compared to leptin-stimulated group.

**Figure 2:**
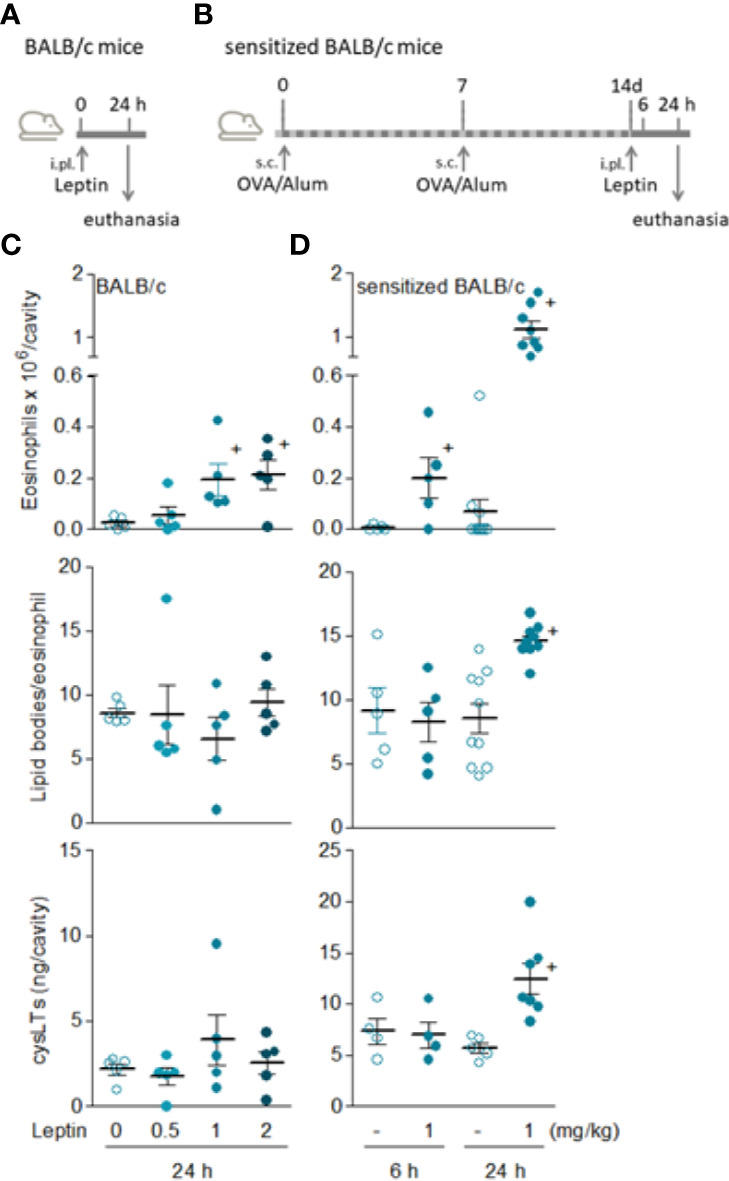
*Leptin induces both in vivo eosinophil migration and activation in sensitized BALB/c mice.* **(A, B)** show, respectively, brief schematic representations of pleurisy models induced by i.pl. injection of leptin in naïve [data shown in **(C)**] and sensitized [data shown in **(D)**] BALB/c mice employed in this study. In **(C)**, naïve BALB/c mice received i.pl. injection with different concentrations of leptin (0.5, 1 and 2 mg/kg) and pleural fluids were collected within 24 h. In **(D)**, sensitized BALB/c mice received i.pl. injection of leptin (1 mg/kg) and pleural fluids were collected within 6 or 24 h, as indicated. Analyses include pleural eosinophil counts evaluated in cells stained by May-Grünwald-Giemsa (upper panels), numbers of cytoplasmic lipid bodies counted within osmium-stained pleural eosinophils (middle panels) and cysLTs levels found in cell-free pleural fluids measured by specific EIA kit (bottom panels). Values are expressed as the mean ± SEM (experiments were repeated at least once). ^+^ *p* < 0.05 compared to control group.

Eosinophils express leptin receptors, whose direct activation *in vitro* induces mobility, rapid assembly of cytoplasmic lipid bodies (also known as lipid droplets) as well as eicosanoid synthesis (16). Firstly, we aimed to establish whether leptin is capable to trigger an eosinophilic inflammatory response *in vivo*, comprising both cell recruitment and activation. As shown in **Figures 1**, **2**, leptin was able to elicit eosinophil migration into its site of administration. In naïve C57BL/6 mice leptin triggered peritoneal eosinophil accumulation within 24 h in a dose-dependent manner (**Figure 1B**); but not in a selective fashion, since neutrophils also accumulate in response to leptin administration (8). Such effect is dependent on the PI3K/mTOR activation, known to be downstream the leptin receptor signaling. As shown in **Figures 1C, D**, respectively, either pharmacological treatment with rapamycin or the use of PI3Kγ^−/−^ mice impaired leptin-induced eosinophil recruitment. Of note, PI3Kγ^−/−^ mice show no defects on bone marrow production or blood availability of eosinophils (33).

Although significant and reproducible, the discreet phenomenon achieved by leptin in naïve C57BL/6 mice demanded experimental adjustments to allow mechanistic studies. Aiming at a more prominent reaction which would enable characterization of cellular and molecular mechanisms involved in leptin-induced eosinophil inflammation *in vivo*, we evaluated the profile of leptin administration in BALB/cBALB/c mice (**Figure 2A**)—a mouse strain known to be more predisposed to mount eosinophil-enriched inflammatory responses (34, 35). Nevertheless, in naïve BALB/cBALB/c mice just a small eosinophilia of about 2 × 10^5^ eosinophils per cavity (similar magnitude observed in naïve C57BL/6 mice) was observed at sites of leptin administration (0.5, 1, or 2 mg/kg; i.pl.) within 24 h (**Figure 2C**). Also, leptin-triggered pleural eosinophil population from naïve BALB/c mice did not display signs of cellular activation; neither lipid body biogenesis nor LTC_4_ synthesis (**Figure 2C**). In order to favor a robust eosinophil influx in conjunction with cellular activation of infiltrating eosinophils, we moved to a strategy previously described for other eosinophilic stimuli using a protocol of pre-sensitization of BALB/c mice (**Figure 2B**) (28). As shown in **Figure 2D**, the leptin injection (1mg/kg; i.pl.) into previously sensitized BALB/c mice induced: (i) a modest but significant eosinophil accumulation as soon as 6 h after leptin stimulation; (ii) a major pleural eosinophilia within 24 h, that reached a magnitude about 6 times higher than that triggered by leptin in naïve BALB/c mice; (iii) a selective pleural eosinophilic phenomenon, since no pleural neutrophilia or increased numbers of mononuclear cells were found in parallel to 6 and 24 h eosinophil influx (**Supplementary Figure S1**); (iv) a parallel drop in circulatory eosinophil numbers noted 24 h after leptin stimulation that was not accompanied by alterations in bone marrow eosinophil population (**Supplementary Figure S2**), indicating that pleural eosinophils were mobilized from the blood pool; and more remarkable, (v) clear activation of pleural infiltrating eosinophils.

By employing an *in vitro* functional approach, we demonstrated that the sensitization of BALB/c mice does not modulate eosinophil chemotactic response, since the magnitude of eotaxin-, PGD_2_-, or leptin-induced *in vitro* chemotaxis of eosinophils recovered from naïve *versus* sensitized animals were not significantly different (**Supplementary Figure S3**). While this finding does not explain why sensitization is required to leptin-driven robust eosinophilic response in BALB/c mice (**Figure 2**), it indicates that the difference may lay, for instance, on *in situ* changes on resident cell populations, like the small pleural mast cell hyperplasia raised by sensitization (described below). In addition, systemic increases in eosinophil population availability could also contribute to it, since in sensitized BALB/c mice both bone marrow and circulating eosinophil populations appear to be slightly (although not statistically significantly) increased in comparison to basal numbers found in naive BALB/c mice (**Supplementary Figure S2**).

**Figure 3:**
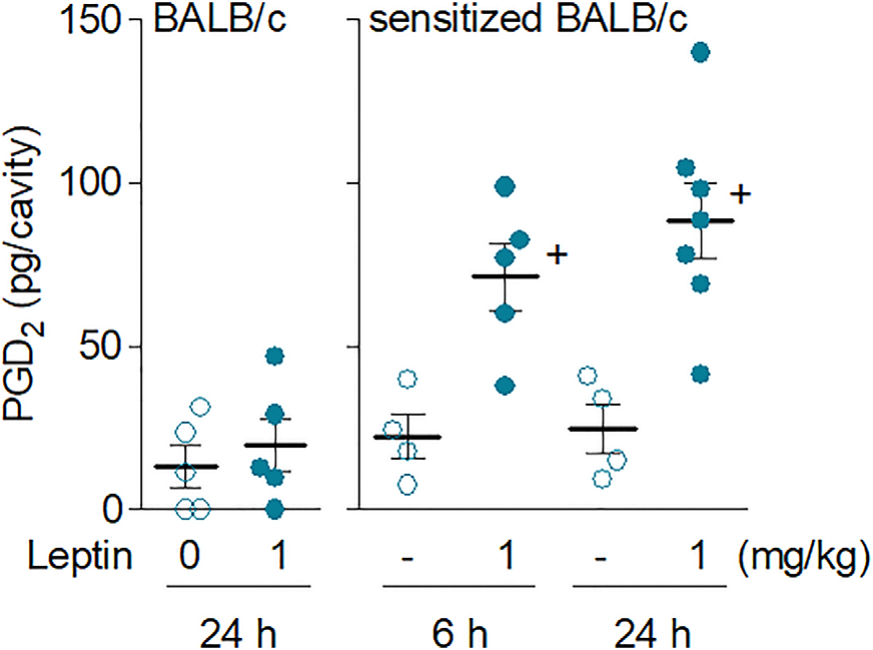
*Leptin administration in vivo elicits rapid secretion of PGD_2_ in sensitized BALB/c mice.* Leptin (1 mg/kg; i.pl.) was injected in naïve (left panel) or sensitized BALB/c mice (right panel). PGD_2_ levels found 6 or 24 h (as indicated) after leptin stimulation in pleural fluids were evaluated as described in Methods. Values are expressed as the mean ± SEM. ^+^ *p* < 0.05 compared to control group.

Exclusively detected in sensitized BALB/c mice, the *in vivo* eosinophil activation elicited by leptin was characterized by increased biogenesis of cytoplasmic lipid bodies within infiltrating eosinophils and enhanced synthesis of LTC_4_ in 24 h (**Figure 2D**). Of note, although LTC_4_ levels were found elevated within 24 h in parallel to the intense eosinophilic reaction triggered by leptin stimulation, no production was found within 6 h— when leptin-elicited pleural eosinophilia was still negligible and the few infiltrating eosinophils showed no sign of cellular activation displaying basal intracellular numbers of lipid bodies (**Figure 2C**). On the other hand, by analyzing other soluble mediators potentially produced within 6 h and therefore prior to the establishment of leptin-induced eosinophilic inflammation at 24 h, we detected increased amounts of PGD_2_ in pleural lavage fluids of sensitized BALB/c mice (**Figure 3**). Leptin-induced pleural PGD_2_ levels remained elevated until 24 h after leptin stimulation in sensitized, but not in naïve BALB/c mice (**Figure 3**), another difference that may represent one of the main mechanisms responsible for the robust eosinophilic response displayed by sensitized in contrast to naïve BALB/c mice.

Sensitized BALB/c mice, besides PGD_2_, also displayed augmented amounts of the chemokine CCL-5 in pleural lavage fluids as early as 6 h after *in vivo* stimulation with leptin (from 14.7 ± 4.9 to 42.3 ± 5.4 pg/ml in control and leptin-stimulated sensitized BALB/c mice, respectively; mean ± SEM of five animals per group; + *p* < 0.05 compared to control group). Even in peritoneal fluids of the naïve C57BL/6 mice, leptin increased CCL5 levels (from 8.3 ± 0.1 to 15.5 ± 2.6 pg/ml in control and leptin-stimulated C57BL/6 mice; respectively; mean ± SEM of at least five animals per group; + *p* < 0.05 compared to control group) and TNFα (8) preceding the minor eosinophilia triggered by leptin in these animals. Taken together, these data suggest that in order to induce eosinophil influx, leptin may first activate one or more resident cell types.

Mast cells emerge as the most plausible cellular candidate to be the target of direct activation by injected leptin, inasmuch as (i) mast cells are prominent resident cells in both peritoneal and pleural cavities; (ii) the number of resident mast cells augments marginally, but significantly, in pleural spaces of sensitized BALB/c mice—0.3 ± 0.06 *versus* 0.1 ± 0.05 × 10^6^ mast cells/cavity in sensitized *versus* naïve BALB/c mice, respectively (mean ± SEM of five animals; *p* < 0.05 compared to naïve animals); (iii) mast cells are well-established regulators of eosinophil migration in a variety of inflammatory conditions (20); (iv) mast cells are known to express leptin receptors (5, 22); (v) TNFα is known to be stored as a preformed cytokine within mast cell granules and therefore are ready for rapid secretion by degranulation (36); and more important and specific (vi) mast cells are acknowledged as the main cellular source of PGD_2_ which is one of the strongest eosinophil chemotactic mediator identified yet (21), also capable to trigger eosinophil activation characterized by lipid body biogenesis and LTC_4_ synthesis (16).

It is worth mentioning that pleural macrophages were our first candidates, rather than pleural mast cells, as the orchestrators of leptin-driven eosinophilic response in sensitized BALB/c mice. However, we focused our studies on resident mast cell population, first because no change on numbers of pleural mononuclear cells was found in leptin-stimulated sensitized BALB/c mice (**Supplementary Figure S1**). More important and exactly alike i.pl. administration of PGD_2_ in sensitized BALB/c mice (28), i.pl. administration of leptin failed to induce lipid body formation within pleural mononuclear cells (data not shown)—a feature consistent with lack of local macrophage activation and enhanced eicosanoid generation. Of note, leptin is known to trigger activation of C57BL/6 peritoneal macrophages, characterized by induced *in vivo* assembly of new highly functional lipid bodies (9). Further studies would be important to evaluate possible interactions between mast cells and macrophages in the C57BL/6 peritoneal cavity.

Altogether, these data consolidates pleurisy in sensitized BALB/c mice as a better suited experimental model to further mast cell/eosinophil studies under leptin stimulation *in vivo*.

### Leptin-Induced Eosinophil Influx and Activation Are Dependent on Resident Mast Cells and PGD_2_ Synthesis

**Figure 4:**
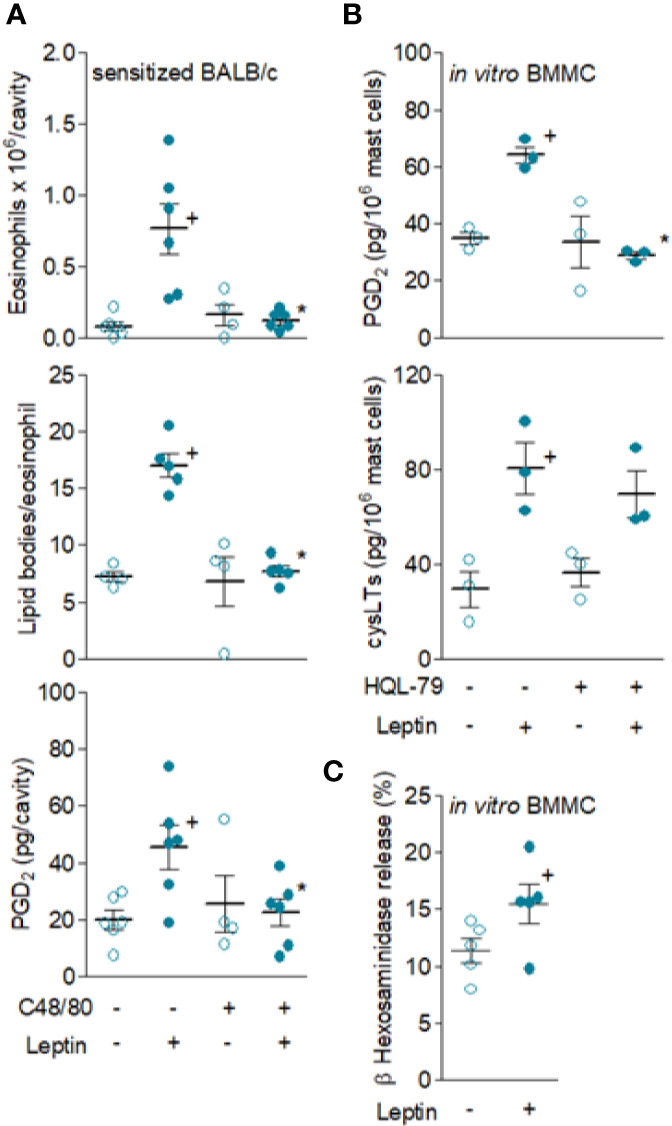
*Resident mast cells are leptin targets and are responsible for in vivo leptin-induced eosinophil migration and activation.* In **(A)**, sensitized BALB/c mice were pre-treated with degranulating mast cell agent compound 48/80 and stimulated with leptin (1 mg/kg; i.pl.). Pleural fluids were collected after 24 h of leptin stimulation. Analyses include pleural eosinophil counts evaluated in cells stained by May-Grünwald-Giemsa (upper panels), PGD_2_ levels found in cell-free pleural fluids measured by specific EIA kit (middle panels) and numbers of cytoplasmic lipid bodies counted within osmium-stained pleural eosinophils (bottom panels). Values are expressed as the mean ± SEM (experiments were repeated at least once). ^+^ *p* < 0.05 compared to control group and *p < 0.05 compared to leptin-stimulated group. In **(B, C)**, mouse *in vitro* differentiated bone marrow-derived mast cells (BMMC) were pre-treated or not (as indicated) with HQL-79 for 30 min and then stimulated with 50 nM of leptin for 1 h. **(B)** shows PGD_2_ and cysLTs levels found in BMMC supernatants quantified by specific EIA kits. **(C)** shows BMMC degranulation by means of detection of extracellular activity of the intragranular enzyme β-hexosaminidase. Values are expressed as the mean ± SEM of different *in vitro* differentiation mast cells cultures (as indicated). ^+^ *p* < 0.05 compared to control cells.

In order to study the involvement of resident mast cells on leptin-induced eosinophil migration, we assessed the effect of experimental strategies that selectively interfered with *in vivo* mast cell activity as well as reduced synthesis of the distinctive eosinophilotactic mast cell-derived mediator PGD_2_. The mast cell degranulating agent compound 48/80 did inhibit leptin-induced eosinophil influx and activation detected 24 h after stimulation of sensitized BALB/c mice (**Figure 4A**), since pleural eosinophil numbers and lipid body content within infiltrating eosinophils were reduced. Of key interest and supporting the role of mast cells-derived PGD_2_ on leptin-induced eosinophilic response, compound 48/80 treatment also reduced leptin-induced PGD_2_ amounts detected within pleural spaces of sensitized BALB/c mice (**Figure 4A**). The role of mast cells as the direct cellular target of leptin stimulation *in vivo* was reinforced by the observation of leptin being also capable to trigger both *in vitro* degranulation, measured by release of granule protein marker β-hexosaminidase enzyme from BMMC (**Figure 4C**), as well as discreet but significant and reproducible *in vitro* PGD_2_ and LTC_4_ synthesis (**Figure 4B**). As also shown in **Figure 4B**, pretreatment with HQL-79, a selective inhibitor of PGD synthase, impaired the acute (detected within 1 h) leptin-induced PGD_2_ synthesizing activity within BMMC, without affecting LTC_4_. Moreover and as previously demonstrated for eosinophils (16), leptin-stimulated BMMC pre-treated with the PI3K inhibitor LY294002 (10 μM) exhibited decreased LTC_4_ synthesis (supernatant levels of cysLTs dropped from 80.4 ± 10.8 to 39.3 ± 12.0 pg/ml in non-treated and LY294002-treated leptin-stimulated BMMC, respectively; mean ± SEM of three *in vitro* differentiated mast cell cultures; + *p* < 0.05 compared to non-treated group). This data indicates that leptin is able to activate cannonical signaling pathways in mast cells that culminate in the generation of relevant inflammatory mediators.

**Figure 5:**
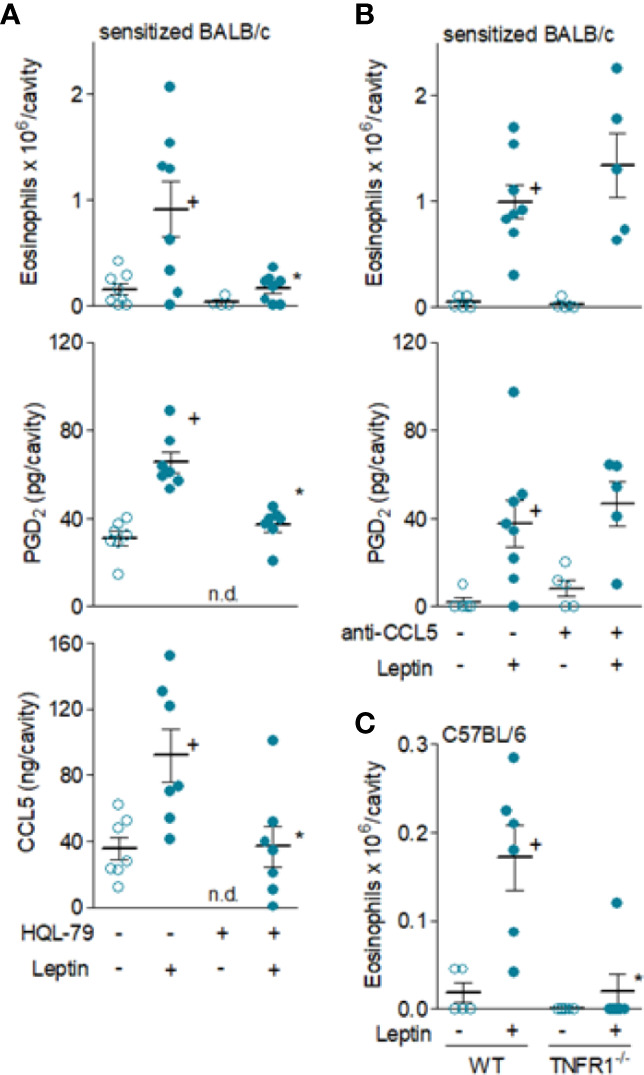
*Leptin-induced in vivo eosinophil migration is mediated by PGD_2_ and TNFα, but not by CCL5.* In **(A, B)**, sensitized BALB/c mice received i.pl. injection of leptin (1 mg/kg) and pleural fluids were collected within 24 h. While in **(A)**, animals were pre-treated with HQL-79 (1 mg/kg, i.p.), in **(B)** mice were pre-treated with a neutralizing anti-CCL5 antibody (10 µg/cavity, i.pl.). In **(C)**, naïve wild type or TNFR1 deficient C57BL/6 mice received i.p. injection of leptin (1 mg/kg) and peritoneal fluids were collected within 24 h of stimulation. As indicated, analyses include pleural eosinophil counts evaluated in cells stained by May-Grünwald-Giemsa as well as PGD_2_ and CCL-5 levels found in cell-free pleural fluids measured by specific EIA and ELISA kits (middle panels). Values are expressed as the mean ± SEM (experiments were repeated at least once). ^+^ *p* < 0.05 compared to control group and *p < 0.05 compared to leptin-stimulated group. n.d.; not determined.

PGD_2_ is a major mast cell product that appears to be critical for the pathogenesis of a variety of eosinophilic disorders (37, 38), mostly because it is a potent chemoattractant for eosinophils both *in vitro* (39, 40) and *in vivo* (28, 41, 42). Of note, and precisely as observed here for leptin, PGD_2_’s ability to induce local eosinophilia in BALB/c mice also depends on specific experimental strategies to create a proper PGD_2_-sensitive environment (34, 43, 44), such as the sensitization of BALB/c mice protocol employed elsewhere (45) and here. Therefore, to investigate the role of PGD_2_ in eosinophil influx triggered by leptin administration, sensitized BALB/c mice were pretreated with HQL-79. As expected, concurrent to inhibition of leptin-induced PGD_2_ synthesis *in vivo*, HQL-79 also inhibited leptin-induced eosinophil influx detected 24 h after i.pl. injection in sensitized BALB/c mice (**Figure 5A**), showing that endogenous PGD_2_ produced during leptin-elicited reaction has an important role in the *in vivo* eosinophil migration. Interestingly, HQL-79 pretreatment also reduced the leptin-induced CCL-5 found within 24 h in sensitized BALB/c mice (**Figure 5A**). This indicates that PGD_2_ may be a central molecule also capable of controlling the secretion of additional mediators from leptin-elicited eosinophilic responses *in vivo*. In line with such autocrine/paracrine activity of PGD_2_ shown here for *in vivo* leptin-stimulated mast cells, we have previously observed some PGD_2_/CCL-5 loop for *in vitro* leptin-stimulated eosinophils (16).

Likewise PGD_2_, it is well established that CCR3 activation by some CC chemokines, including CCL5, promotes potent eosinophil chemoattraction and mast cells have been reported to produce and release CCL5 (46). As shown in **Figure 5B**, the pre-treatment with a neutralizing antibody against mouse CCL5 failed to interfere with eosinophil accumulation triggered by leptin administration in sensitized BALB/c mice, therefore indicating that those earlier elevated levels of CCL5 found in leptin-elicited inflammatory sites do not participate in eosinophil migration. Differently, leptin-driven enhanced TNFα levels—which precede eosinophil influx—appear to contribute to it, as evidenced by the lack of eosinophil migration in TNFR1 deficient mice (C57BL/6 background) after leptin injection (**Figure 5C**). This result indicates that activation of TNFα receptor TNFR1 by endogenous TNFα may contribute to leptin-induced eosinophil migration *in vivo.*

### PGD_2_ and CCL5 Mediate *In Situ* Eosinophil Activation Triggered by Leptin *In Vivo*

Recently, we have identified leptin as a novel mediator capable of activating the biogenesis of lipid bodies and enhanced LTC_4_ production within eosinophils (16), suggesting that part of the *in vivo* mechanisms of leptin-induced lipid body-driven LTC_4_ production would be due to a direct stimulatory effect of leptin on recruited eosinophils. However, as virtually no resident eosinophils are present in the pleural or peritoneal cavities neither in naïve nor sensitized mice (not shown), the leptin-induced cellular activation of infiltrating eosinophils seem to be an indirect phenomenon. Therefore, the initial response to leptin *in vivo* may depend on direct activation of resident cells and triggered by mediators produced upon exogenous leptin stimulation.

**Figure 6:**
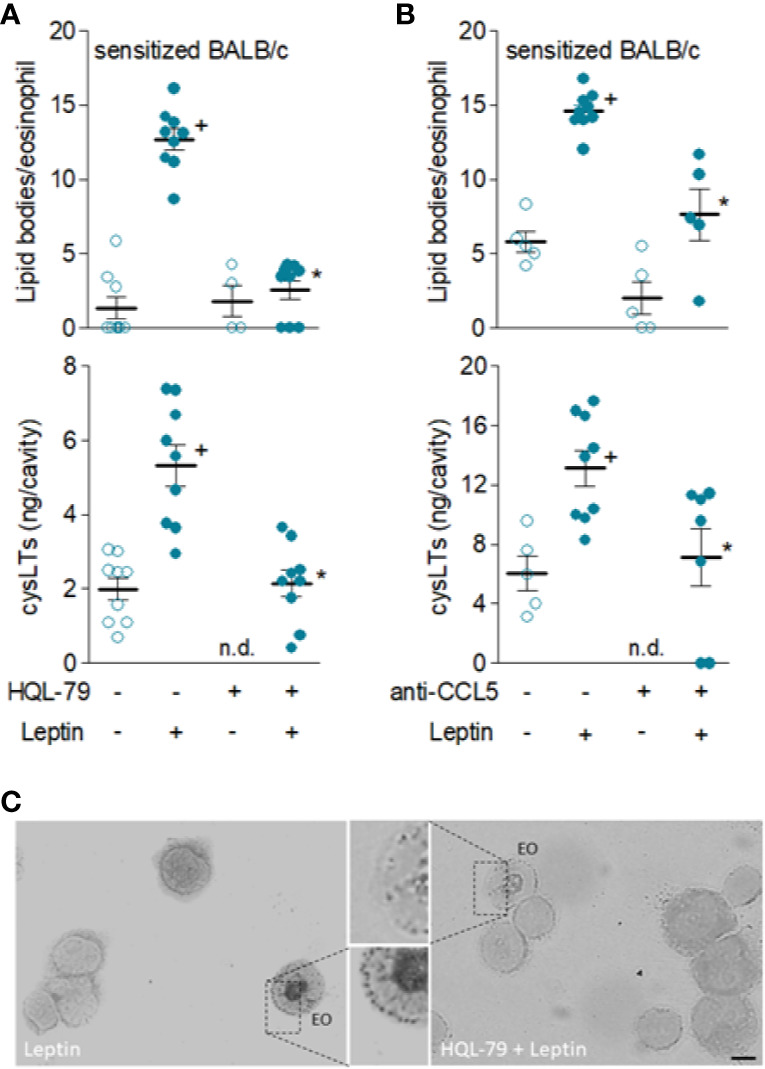
*Leptin-induced in vivo eosinophil activation in sensitized BALB/c mice is mediated by PGD_2_ and CCL5.* Sensitized BALB/c mice received i.pl. injection of leptin (1 mg/kg) and pleural fluids were collected within 24 h of stimulation. While in **(A)**, animals were pre-treated with HQL-79 (1 mg/kg, i.p.), in **(B)** mice were pre-treated with a neutralizing anti-CCL5 antibody (10 µg/cavity, i.pl.). Analyses include numbers of cytoplasmic lipid bodies counted within osmium-stained pleural eosinophils (upper panels) and cysLTs levels found in cell-free pleural fluids measured by specific EIA kit (bottom panels). **(C)** shows representative images of osmium-stained pleural cells recovered from leptin-stimulated sensitized BALB/c mice which were pre-treated or not (as indicated) with HQL-79. Values are expressed as the mean ± SEM (experiments were repeated at least once). ^+^ *p* < 0.05 compared to control group and *p < 0.05 compared to leptin-stimulated group. n.d.; not determined. EO; eosinophil. Scale bar = 5 µm.

Among very few other specific stimuli (21), PGD_2_ and CCL5 are known to directly promote activation of *de novo* formation of lipid bodies and LTC_4_ synthesis by eosinophils both *in vitro* and *in vivo* (28, 47). Here, several of our findings confirm that leptin-induced eosinophil activation *in vivo* appears to be dependent on mast cell-derived PGD_2_-driven paracrine activity in sensitized BALB/c mice, since (i) increased PGD_2_ levels detected as early as 6 h (**Figure 3B**) of leptin administration are kept elevated for at least 24 h in eosinophilic inflammatory site of leptin-challenged sensitized BALB/c; (ii) pretreatment with compound 48/80 reduced *in vivo* leptin-induced production of PGD_2_ (**Figure 4A**) in sensitized BALB/c mice; (iii) mast cell degranulating agent compound 48/80 blocked leptin-induced assembly of new cytoplasmic lipid bodies within recruited eosinophils as detected 24 h after leptin stimulation of sensitized BALB/c mice (**Figure 4A**); and, more significantly, (iv) specific inhibition of PGD_2_ synthesis by HQL-79 disrupted both biogenesis of cytoplasmic lipid bodies and LTC_4_ synthesis within recruited eosinophils in leptin-stimulated sensitized BALB/c mice (**Figures 6A, C**).

Remarkably, even though CCL5 did not appear to participate on the induction of leptin-elicited eosinophil migration *in vivo*, it does mediate leptin-induced *in vivo* eosinophil activation, inasmuch as the pre-treatment with the neutralizing antibody to CCL5 did inhibit leptin-driven assembly of new lipid bodies within recruited eosinophils and LTC_4_ synthesis (**Figure 6B**).

## Discussion

It is now broadly accepted that eosinophils are fundamental keepers of adipose tissue homeostasis, capable of thwarting obesity-related metabolic syndrome (18, 48). As adipose sentinel cells which express leptin receptors, eosinophils may be under constant leptin stimulation. *In vitro* studies unveiled leptin as a wide-ranging stimulus for eosinophils, eliciting eosinophil kinesis, cytokine secretion, lipid body biogenesis, and the highly regulated events of eicosanoid synthesis (13–16). However, in contrast to the well documented leptin-induced *in vitro* eosinophil activation, studies addressing whether leptin affects eosinophil population *in vivo* are scarse.

Here, we demonstrated the capability of leptin in triggering eosinophilic inflammation *in vivo* in naïve C57BL/6 or BALB/c mice. Interestingly, the leptin induced eosinophil migration was more prominent in sensitized BALB/c mice. In addition, leptin was successful in triggering activation of the infiltrating eosinophils only in pre-sensitized BALB/c mice. Evaluation of these two mouse backgrounds are currently of key relevance; inasmuch as recent reports employing BALB/c mice have defied the paradigm derived from C57BL/6 studies that eosinophils are absent from obese adipose tissue, while revealed even far-reaching protective functions of adipose tissue eosinophils (18, 48).

It has been shown that, no matter the metabolic state displayed by the adipose micro-environment, both the homeostatic functions of eosinophils and the general mechanisms regulating eosinophil presence within adipose tissue are alike; including the mediation by a common tripod of type 2 innate lymphoid cells (ILC2s), IL-5, and eotaxin (4, 5). However, the multiplicity of adipose micro-environmental factors potentially capable of establishing adipose eosinophilia and/or properly activate eosinophil functions is far more abundant and obviously not restricted to these three key players, and as shown here, may involve leptin as an additional molecular modulator.

It seems definitive that eosinophilia is a hallmark of lean adipose niches and that eosinophils counteract the obesity related chronic inflammation (18, 48). This knowledge is derived from a large body of data using C57BL/6 mouse experimental models of obesity which established the reduction of homeostatic eosinophils in adipose tissue undergoing metabolic syndrome (4, 5). Nevertheless, Lee et al. found contrasting results in adipose tissue eosinophils of BALB/c mice under high-fat diet (18). The adipose tissue eosinophils appear to increase in numbers, rather than vanish. Such difference in eosinophil dynamics has been immediately attributed to the well-established feature of preferential responsive shifts toward Type 1 *versus* Type 2 immune profiles by C57BL/6 *versus* BALB/c mice, respectively (49). Consistent with this proposition, we have shown here a more robust leptin-stimulated eosinophilic response seen in BALB/c mice which were submitted to previous sensitization—an experimental procedure known to promptly evoke intense Type 2 response characterized by eosinophilic outcomes. While we did not focus on characterizing the mechanisms explaining why sensitization of BALB/c mice promotes the establishment of a more intense, selective and whole (migration *plus* activation) leptin-driven eosinophilic response, we have identified at least three factors that in association may contribute to the overall more robust pleural eosinophilic reaction. These factors are: (i) an increased number of mast cells residing in pleural cavities; (ii) an increased availability of bone marrow and blood eosinophils; and (iii) the local production of PGD_2_. Of note, recent clinical results showed similar results to BALB/c mice with increased adipose eosinophilia in a group of obese patients (50); therefore indicating that BALB/c models may reproduce better adipose tissue-related human eosinophilic events.

According to the study by Lee and coworkers (18), rather than the size of eosinophil population within adipose niche, the profile of eosinophil activation emerges as the central element of tissue function. Overall, these authors reported that, even though eosinophils accumulate in adipose tissue of obese BALB/c mice, they display homeostatic functions as proposed for the lean adipose tissue (18). Therefore, evaluating *in vivo* eosinophil activation, instead of the exclusive assessment of their migration pattern, was germane here. So far, the acknowledged mechanism of eosinophil homeostatic roles lays essentially on activation of eosinophil secretory activity of immune-modulator proteins, mostly cytokines like IL-4 and IL-13 (48, 51, 52). However, eosinophils have a much diverse secretory capability, which is not limited to cytokine secretion, but eosinophils are remarkable sources of multifunctional lipid mediators. Within physiologically stimulated eosinophils, bioactive lipids from arachidonic acid metabolism are synthesized primarily in cytoplasmic lipid bodies (53, 54). Of note, cytoplasmic lipid body numbers are characteristically increased within eosinophils following *in vitro* stimulation, as well as in *in vivo* inflammatory disorders, and are used as a marker of eosinophil activation (55). Besides the previously reported direct activation of lipid body biogenesis *in vitro* within eosinophils by leptin (16), we demonstrated that leptin administration in sensitized BALB/c mice promotes formation of new lipid bodies within the eosinophils recruited to the leptin ability to evoke eosinophil activation *in vivo*.

The assembly of these highly active organelles within eosinophils is often linked to intense synthesis of eicosanoids, notably LTC_4_, which was produced upon *in vivo* stimulation with leptin. LTC_4_ may drive adipose homeostasis since it (i) potentiates IL-5 release from ILC2s (56) and (ii) elicits IL-4 secretion from eosinophils (57). Noteworthy, we have recently demonstrated that leptin prompts LTC_4_ synthesis within the newly formed cytoplasmic lipid bodies in human and mouse eosinophils *in vitro* (16). Besides LTC_4_, increased leptin levels triggered by adipose tissue dysfunction, may also unbalance eosinophil lipid body-compartmentalized synthesis of other eicosanoids known to interfere with adipogenesis and inflammation (58, 59), such as the prostanoids PGE_2_ and PGD_2_ (16). Eosinophil lipid bodies are highly active intracellular sites of eicosanoid synthesis (54, 55) and may have important roles, not only in the adipose tissue physiology, but also in metabolic disease states, therefore indicating that further studies on the potential impacts of activation of eosinophilic lipid bodies on adipose tissue are germane.

Considering both leptin receptor expression by eosinophils (13) and *in vitro* leptin capability to activate eosinophils (14), one could assume that *in vivo* leptin-induced eosinophil activation described here is a consequence of direct stimulation of leptin receptors on eosinophil surface. Without precluding a partial direct effect of leptin onto eosinophils in the overall leptin-driven eosinophilic response *in vivo* described here, as eosinophils are not resident cells within the pleural cavity and, therefore, absent at the moment of leptin administration; leptin ability to induce *in vivo* eosinophil activation may mostly depend on indirect components. Among the various resident cellular targets for leptin stimulation *in vivo*, we identified mast cells as a key cell population. Importantly, our results identify mast cells as modulators of leptin-driven eosinophilic reaction are in line with recent postulations that adipose tissue resident mast cells or the obesity-related increased mast cell population are modulators of adipose tissue homeostasis and inflammation (60, 61).

Several pieces of evidence indicate that mast cells secretory activity is involved in leptin-elicited eosinophil influx and lipid body-driven LTC_4_ synthesis *in vivo*, since: (i) the understanding that mast cells express active leptin receptors (22, 62); (ii) the ability of leptin to directly arouse mast cell secretory activities, as showed here and by others (63); (iii) the leptin-elicited increased local levels of classical mast cell-derived eosinophil-relevant stimuli which precede eosinophil arrival but may remain elevated during installed eosinophilia; (iv) the *in vivo* inhibitory effect achieved by selectively targeting mast cells with the compound 48/80; and (v) the paracrine role of classical mast cell-derived molecules in the leptin-driven induction of eosinophil recruitment and activation. Among mast cells-derived molecules, PGD_2_ may represent one of the most classical and unambiguous products (37). Here, PGD_2_ synthesis in mast cells was promoted by leptin both *in vitro* and *in vivo*. Moreover, increased local levels of mast cell-derived PGD_2_ were crucial for both influx and activation of eosinophils induced by leptin. Besides a potent eosinophilotactic factor, this prostanoid is undeniably also a known eosinophil activator (64). In an analogous manner to leptin, PGD_2_ is able to trigger lipid body biogenesis and compartmentalized LTC_4_ synthesis both *in vitro* within human and mice eosinophils, as well as *in vivo* only in sensitized BALB/c mice.

In addition to PGD_2_, the mechanisms involved in leptin-induced lipid body-driven LTC_4_ synthesis also include CCL5 as another mediator of the *in vivo* phenomenom in sensitized BALB/c mice. Strikingly, even though CCL5 is a chemokine capable of promoting both eosinophilotaxis and LTC_4_ synthesis within newly formed lipid bodies (47), it did not participate in eosinophil migration while mediated *in vivo* eosinophil activation triggered by leptin. Therefore, we concluded that although both eosinophilic events depend on paracrine activities mediated by mast cell-derived PGD_2_, eosinophil migration and activation triggered by leptin *in vivo* seem to be independent phenomena mediated by different sets of locally produced factors. Additional chemotactic mediators may synergize with PGD_2_ to promote leptin-induced eosinophil. Of note, another classical mast cell-derived molecule addressed here—the pro-inflammatory cytokine TNFα—appears to also contribute to eosinophil accumulation in response to leptin stimulation, even though it is not recognized as a direct eosinophil chemotactic molecule.

We have previously shown that leptin is capable of eliciting pro-adipogenic and pro-inflammatory signaling in adipocytes, as well as eicosanoid production in macrophages, neutrophils, eosinophils (8, 16, 65). Here, by further evidencing the capacity of leptin to modulate the interplay between eosinophils and mast cells, we suggest leptin as a key factor of the inflammation-modulated homeostasis in the adipose tissue.

By extrapolating the findings presented here to adipose tissue, one can speculate that leptin continuously produced by mature adipocytes may elicit local synthesis of bioactive PGD_2_ by PGDS-expressing resident cells, such as mast cells and eosinophils. Functioning in a paracrine fashion within adipose tissue, locally produced PGD_2_ may trigger a variety of adipose housekeeping mechanisms with potential impact on metabolic syndrome evasion, including (i) down-regulation of leptin production (66), secretion of Type 2 cytokines IL-5 as well as IL-4 by ILC2s (67, 68), activation of adipose eosinophils (16) and/or polarization of macrophages toward a M2 anti-inflammatory state (69).

**Figure 7:**
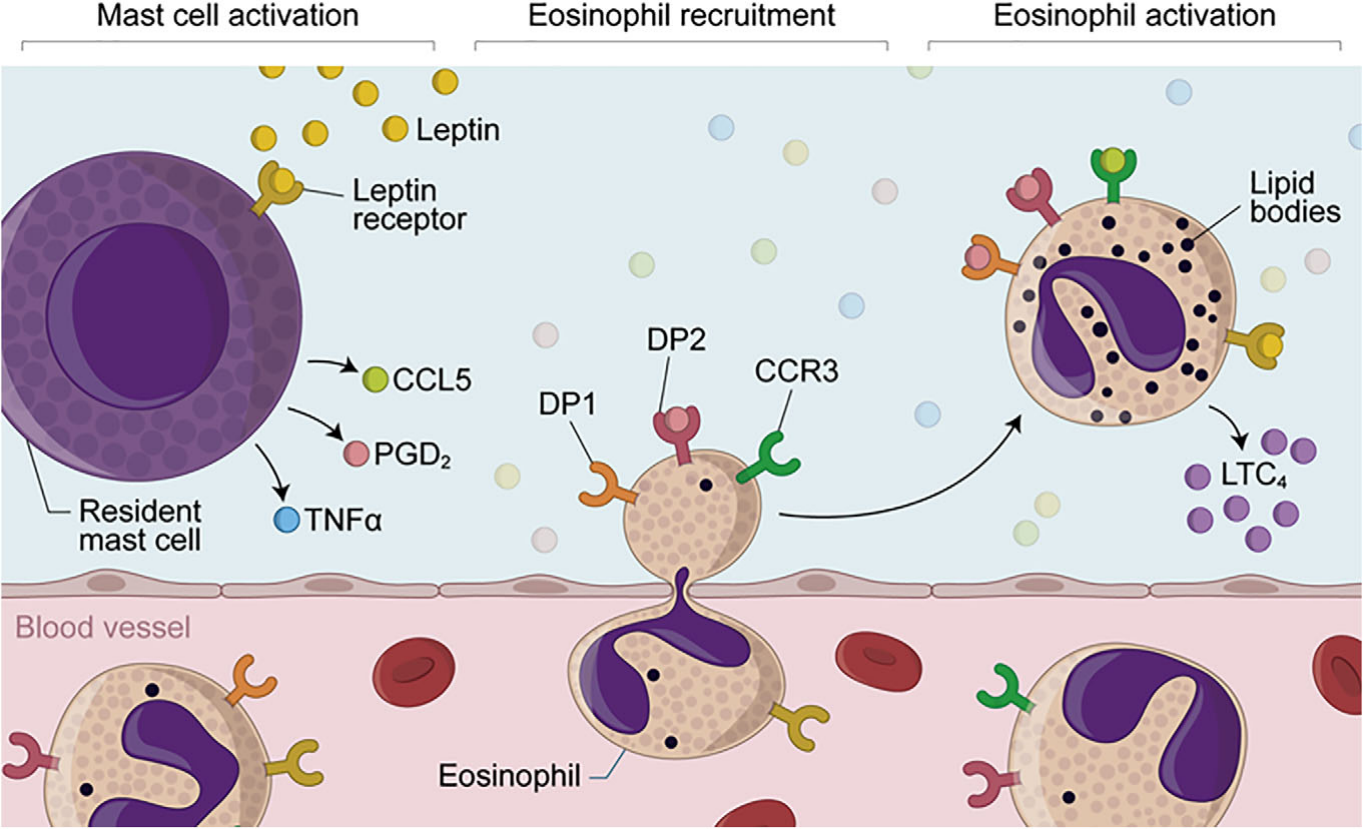
*Proposed mechanisms of leptin-induced in vivo eosinophilic inflammation mediated by mast cell-derived PGD_2_.* Eosinophilic inflammation triggered by *in vivo* stimulation with leptin depends on sequential events, including (i) initial activation of resident mast cell secretory activity; (ii) increased local levels of TNFα, CCL5 and PGD_2_; (iii) induction of eosinophil migration by TNFα and PGD_2_ and then; (iv) cellular activation of recruited eosinophils, characterized by lipid body biogenesis and LTC_4_ synthesis. While mast cell-derived PGD_2_ may induce eosinophil influx *via* activation of its chemotactic DP2 receptor which is highly expressed on eosinophils, leptin-driven *in situ* eosinophil activation is mediated by both CCL5 and PGD_2_ throughout simultaneous activation of CCR3 and PGD_2_ receptors DP1 and DP2— whose downstream signaling are known to culminate with *in vitro* lipid body-compartmentalized synthesis of LTC_4_ within leptin-stimulated eosinophils.

Collectively, our data unveiled a scenario where leptin interacts with resident mast cells, which in turn govern eosinophil numbers as well as set up a proper *in situ* eosinophil activation (for proposed mechanisms see **Figure 7**). Moreover, PGD_2_ emerges as an important player of leptin-mediated cell crosstalk between mast cells and eosinophils, which culminates with LTC_4_ production. Therefore, it seems clear that further investigations addressing eosinophil-driven homeostatic roles in adipose tissue must include studies on the role of leptin-driven immune-modulatory lipid mediators in addition to the well-established role of eosinophil-derived cytokines.

## Data Availability Statement

The raw data supporting the conclusions of this article will be made available by the authors, without undue reservation.

## Ethics Statement

The animal study was reviewed and approved by both Animal Use Welfare Committees of Federal University of Rio de Janeiro (CEUA-CCS/UFRJ license number 090/18) and Oswaldo Cruz Institute (CEUA-IOC license number L-011/2015).

## Author Contributions

CM-M and CB-M conceived and designed the study. NA, GS-A, and TL-G designed and performed the experiments, analyzed, and interpreted data. NA, GS-A, TL-G, PB, CC, and BD participated in the data acquisition, analysis, and interpretation. PB, CC, BD, CM-M and CB-M wrote and revised the manuscript. All authors contributed to the article and approved the submitted version.

## Funding

This work was supported by the Fundação de Amparo à Pesquisa do Estado do Rio de Janeiro_Faperj (grants E-26/202.926/2015 to CB-M and E-26/110.374/2014 to CM-M); the Conselho Nacional de Desenvolvimento Científico e Tecnológico (CNPq; grants 453826/2014-8 to CB-M and 306290/2014-6 to CM-M); and Coordenação de Aperfeiçoamento de Pessoal de Nível Superior_CAPES (fellowship to GS-A); PAPES/FIOCRUZ (grant 407442/2012-0 to CM-M), and aboratory financial support from Oswaldo Cruz Institute (IOC/FIOCRUZ) (to CM-M and PB). Disclaimer: The funders had no role in study design, data collection/analysis, decision to publish, or preparation of the manuscript.

## Conflict of Interest

The authors declare that the research was conducted in the absence of any commercial or financial relationships that could be construed as a potential conflict of interest.

## References

[1] Klion AD Rothenberg ME Advances in eosinophilic diseases in 2018. J Allergy Clin Immunol (2019) 144:1490–4. 10.1016/j.jaci.2019.10.010 31655098

[2] Weller PF Spencer LA Functions of tissue-resident eosinophils. Nat Rev Immunol (2017) 17:746–60. 10.1038/nri.2017.95 PMC5783317 28891557

[3] Shah K Ignacio A McCoy KD Harris NL The emerging roles of eosinophils in mucosal homeostasis. Mucosal Immunol (2020) 13(4):574–83. 10.1038/s41385-020-0281-y 32157190

[4] Calco GN Fryer AD Nie Z Unraveling the connection between eosinophils and obesity. J Leukoc Biol (2020) 108(1):123–8. 10.1002/JLB.5MR0120-377R PMC9233491 32170879

[5] Vohralik EJ Psaila AM Knights AJ Quinlan KGR EoTHINophils: Eosinophils as key players in adipose tissue homeostasis. Clin Exp Pharmacol Physiol (2020) 47(8):1495–505. 10.1111/1440-1681.13304 32163614

[6] Larabee CM Neely OC Domingos AI Obesity: a neuroimmunometabolic perspective. Nat Rev Endocrinol (2020) 16:30–43. 10.1038/s41574-019-0283-6 31776456

[7] Francisco V Pino J Campos-Cabaleiro V Ruiz-Fernández C Mera A Gonzalez-Gay MA Obesity, Fat Mass and Immune System: Role for Leptin. Front Physiol (2018) 9:640. 10.3389/fphys.2018.00640 29910742 PMC5992476

[8] Souza-Almeida G D’Avila H Almeida PE Luna-Gomes T Liechocki S Walzog B Leptin Mediates In Vivo Neutrophil Migration: Involvement of Tumor Necrosis Factor-Alpha and CXCL1. Front Immunol (2018) 9:111. 10.3389/fimmu.2018.00111 29467755 PMC5808117

[9] Maya-Monteiro CM Almeida PE D’Avila H Martins AS Rezende AP Castro-Faria-Neto H Leptin induces macrophage lipid body formation by a phosphatidylinositol 3-kinase- and mammalian target of rapamycin-dependent mechanism. J Biol Chem (2008) 283:2203–10. 10.1074/jbc.M706706200 18039669

[10] Procaccini C Pucino V De Rosa V Marone G Matarese G Neuro-endocrine networks controlling immune system in health and disease. Front Immunol (2014) 5:143. 10.3389/fimmu.2014.00143 24778633 PMC3985001

[11] Mancuso P Curtis JL Freeman CM Peters-Golden M Weinberg JB Myers MG Ablation of the leptin receptor in myeloid cells impairs pulmonary clearance of Streptococcus pneumoniae and alveolar macrophage bactericidal function. Am J Physiol-Lung Cell Mol Physiol (2018) 315:L78–86. 10.1152/ajplung.00447.2017 PMC6087898 29565180

[12] Mancuso P Obesity and respiratory infections: does excess adiposity weigh down host defense? Pulm Pharmacol Ther (2013) 26:412–9. 10.1016/j.pupt.2012.04.006 PMC3439591 22634305

[13] Conus S Bruno A Simon H-U Leptin is an eosinophil survival factor. J Allergy Clin Immunol (2005) 116:1228–34. 10.1016/j.jaci.2005.09.003 16337450

[14] Wong CK Cheung PF-Y Lam CWK Leptin-mediated cytokine release and migration of eosinophils: implications for immunopathophysiology of allergic inflammation. Eur J Immunol (2007) 37:2337–48. 10.1002/eji.200636866 17634954

[15] Kato H Ueki S Kamada R Kihara J Yamauchi Y Suzuki T Leptin has a priming effect on eotaxin-induced human eosinophil chemotaxis. Int Arch Allergy Immunol (2011) 155:335–44. 10.1159/000321195 21346363

[16] Amorim NRT Luna-Gomes T Gama-Almeida M Souza-Almeida G Canetti C Diaz BL Leptin Elicits LTC4 Synthesis by Eosinophils Mediated by Sequential Two-Step Autocrine Activation of CCR3 and PGD2 Receptors. Front Immunol (2018) 9:2139. 10.3389/fimmu.2018.02139 30298073 PMC6160734

[17] Calixto MC Lintomen L Schenka A Saad MJ Zanesco A Antunes E Obesity enhances eosinophilic inflammation in a murine model of allergic asthma. Br J Pharmacol (2010) 159:617–25. 10.1111/j.1476-5381.2009.00560.x PMC2828025 20100278

[18] Lee E-H Itan M Jang J Gu H-J Rozenberg P Mingler MK Eosinophils support adipocyte maturation and promote glucose tolerance in obesity. Sci Rep (2018) 8:9894. 10.1038/s41598-018-28371-4 29967467 PMC6028436

[19] Stone KD Prussin C Metcalfe DD IgE, mast cells, basophils, and eosinophils. J Allergy Clin Immunol (2010) 125:S73–80. 10.1016/j.jaci.2009.11.017 PMC2847274 20176269

[20] Robida PA Puzzovio PG Pahima H Levi-Schaffer F Bochner BS Human eosinophils and mast cells: Birds of a feather flock together. Immunol Rev (2018) 282:151–67. 10.1111/imr.12638 PMC5812359 29431215

[21] Luna-Gomes T Bozza PT Bandeira-Melo C Eosinophil recruitment and activation: the role of lipid mediators. Front Pharmacol (2013) 4:27. 10.3389/fphar.2013.00027 23525348 PMC3605515

[22] Taildeman J Pérez-Novo CA Rottiers I Ferdinande L Waeytens A De Colvenaer V Human mast cells express leptin and leptin receptors. Histochem Cell Biol (2009) 131:703–11. 10.1007/s00418-009-0575-3 19241089

[23] Żelechowska P Brzezińska-Błaszczyk E Wiktorska M Różalska S Wawrocki S Kozłowska E Adipocytokines leptin and adiponectin function as mast cell activity modulators. Immunology (2019) 158:3–18. 10.1111/imm.13090 31220342 PMC6700464

[24] Wang Y Yu L Meng G Wang Z Zhou Z Zhang Y Mast cells modulate the pathogenesis of leptin-induced left stellate ganglion activation in canines. Int J Cardiol (2018) 269:259–64. 10.1016/j.ijcard.2018.07.126 30072157

[25] Liu D-R Xu X-J Yao S-K Increased intestinal mucosal leptin levels in patients with diarrhea-predominant irritable bowel syndrome. World J Gastroenterol (2018) 24:46–57. 10.3748/wjg.v24.i1.46 29358881 PMC5757124

[26] Chaldakov GN Fiore M Stankulov IS Hristova M Antonelli A Manni L BDNF, leptin, and mast cells in human coronary atherosclerosis and metabolic syndrome. Arch Physiol Biochem (2001) 109:357–60. 10.1076/apab.109.4.357.4249 11935372

[27] Zheng L Fisher G Miller RE Peschon J Lynch DH Lenardo MJ Induction of apoptosis in mature T cells by tumour necrosis factor. Nature (1995) 377:348–51. 10.1038/377348a0 7566090

[28] Mesquita-Santos FP Vieira-de-Abreu A Calheiros AS Figueiredo IH Castro-Faria-Neto HC Weller PF Cutting edge: prostaglandin D2 enhances leukotriene C4 synthesis by eosinophils during allergic inflammation: synergistic in vivo role of endogenous eotaxin. J Immunol Baltim Md 1950 (2006) 176:1326–30. 10.4049/jimmunol.176.3.1326 16424158

[29] Diaz BL Serra MF Alves AC Pires AL Corrêa FM Cordeiro RS e Silva PM. Alloxan diabetes reduces pleural mast cell numbers and the subsequent eosinophil influx induced by allergen in sensitized rats. Int Arch Allergy Immunol (1996) 111:36–43. 10.1159/000237343 8753842

[30] Diaz BL Fujishima H Kanaoka Y Urade Y Arm JP Regulation of prostaglandin endoperoxide synthase-2 and IL-6 expression in mouse bone marrow-derived mast cells by exogenous but not endogenous prostanoids. J Immunol Baltim Md 1950 (2002) 168:1397–404. 10.4049/jimmunol.168.3.1397 11801681

[31] Murakami M Austen KF Arm JP The immediate phase of c-kit ligand stimulation of mouse bone marrow-derived mast cells elicits rapid leukotriene C4 generation through posttranslational activation of cytosolic phospholipase A2 and 5-lipoxygenase. J Exp Med (1995) 182:197–206. 10.1084/jem.182.1.197 7540649 PMC2192097

[32] Diaz BL Satake Y Kikawada E Balestrieri B Arm JP Group V secretory phospholipase A2 amplifies the induction of cyclooxygenase 2 and delayed prostaglandin D2 generation in mouse bone marrow culture-derived mast cells in a strain-dependent manner. Biochim Biophys Acta (2006) 1761:1489–97. 10.1016/j.bbalip.2006.09.009 PMC1764612 17064958

[33] Pinho V Souza DG Barsante MM Hamer FP De Freitas MS Rossi AG Phosphoinositide-3 kinases critically regulate the recruitment and survival of eosinophils in vivo: importance for the resolution of allergic inflammation. J Leukoc Biol (2005) 77:800–10. 10.1189/jlb.0704386 15860799

[34] Hsieh CS Macatonia SE O’Garra A Murphy KM T cell genetic background determines default T helper phenotype development in vitro. J Exp Med (1995) 181:713–21. 10.1084/jem.181.2.713 PMC2191880 7836924

[35] Jovicic N Jeftic I Jovanovic I Radosavljevic G Arsenijevic N Lukic ML Differential Immunometabolic Phenotype in Th1 and Th2 Dominant Mouse Strains in Response to High-Fat Feeding. PloS One (2015) 10:e0134089. 10.1371/journal.pone.0134089 26218873 PMC4517873

[36] Gordon JR Galli SJ Release of both preformed and newly synthesized tumor necrosis factor alpha (TNF-alpha)/cachectin by mouse mast cells stimulated via the Fc epsilon RI. A mechanism for the sustained action of mast cell-derived TNF-alpha during IgE-dependent biological responses. J Exp Med (1991) 174:103–7. 10.1084/jem.174.1.103 PMC2118884 1829107

[37] Raible DG Schulman ES DiMuzio J Cardillo R Post TJ Mast cell mediators prostaglandin-D2 and histamine activate human eosinophils. J Immunol Baltim Md 1950 (1992) 148:3536–42. 1588043

[38] Matsuoka T Hirata M Tanaka H Takahashi Y Murata T Kabashima K Prostaglandin D2 as a mediator of allergic asthma. Science (2000) 287:2013–7. 10.1126/science.287.5460.2013 10720327

[39] Hirai H Tanaka K Yoshie O Ogawa K Kenmotsu K Takamori Y Prostaglandin D2 selectively induces chemotaxis in T helper type 2 cells, eosinophils, and basophils via seven-transmembrane receptor CRTH2. J Exp Med (2001) 193:255–61. 10.1084/jem.193.2.255 PMC2193345 11208866

[40] Monneret G Gravel S Diamond M Rokach J Powell WS Prostaglandin D2 is a potent chemoattractant for human eosinophils that acts via a novel DP receptor. Blood (2001) 98:1942–8. 10.1182/blood.v98.6.1942 11535533

[41] Spik I Brénuchon C Angéli V Staumont D Fleury S Capron M Activation of the prostaglandin D2 receptor DP2/CRTH2 increases allergic inflammation in mouse. J Immunol Baltim Md 1950 (2005) 174:3703–8. 10.4049/jimmunol.174.6.3703 15749909

[42] Kagawa S Fukunaga K Oguma T Suzuki Y Shiomi T Sayama K Role of prostaglandin D2 receptor CRTH2 in sustained eosinophil accumulation in the airways of mice with chronic asthma. Int Arch Allergy Immunol (2011) 155 Suppl 1:6–11. 10.1159/000327257 21646789

[43] Almishri W Cossette C Rokach J Martin JG Hamid Q Powell WS Effects of prostaglandin D2, 15-deoxy-Delta12,14-prostaglandin J2, and selective DP1 and DP2 receptor agonists on pulmonary infiltration of eosinophils in Brown Norway rats. J Pharmacol Exp Ther (2005) 313:64–9. 10.1124/jpet.104.079079 15590767

[44] Honda K Arima M Cheng G Taki S Hirata H Eda F Prostaglandin D2 reinforces Th2 type inflammatory responses of airways to low-dose antigen through bronchial expression of macrophage-derived chemokine. J Exp Med (2003) 198:533–43. 10.1084/jem.20022218 PMC2194171 12925672

[45] Mesquita-Santos FP Bakker-Abreu I Luna-Gomes T Bozza PT Diaz BL Bandeira-Melo C Co-operative signalling through DP(1) and DP(2) prostanoid receptors is required to enhance leukotriene C(4) synthesis induced by prostaglandin D(2) in eosinophils. Br J Pharmacol (2011) 162:1674–85. 10.1111/j.1476-5381.2010.01086.x PMC3081113 20973774

[46] Mukai K Tsai M Saito H Galli SJ Mast cells as sources of cytokines, chemokines, and growth factors. Immunol Rev (2018) 282:121–50. 10.1111/imr.12634 PMC5813811 29431212

[47] Bandeira-Melo C Phoofolo M Weller PF Extranuclear lipid bodies, elicited by CCR3-mediated signaling pathways, are the sites of chemokine-enhanced leukotriene C4 production in eosinophils and basophils. J Biol Chem (2001) 276:22779–87. 10.1074/jbc.M101436200 11274187

[48] Wu D Molofsky AB Liang H-E Ricardo-Gonzalez RR Jouihan HA Bando JK Eosinophils sustain adipose alternatively activated macrophages associated with glucose homeostasis. Science (2011) 332:243–7. 10.1126/science.1201475 PMC3144160 21436399

[49] Watanabe H Numata K Ito T Takagi K Matsukawa A Innate immune response in Th1- and Th2-dominant mouse strains. Shock Augusta Ga (2004) 22:460–6. 10.1097/01.shk.0000142249.08135.e9 15489639

[50] Moussa K Gurung P Adams-Huet B Devaraj S Jialal I Increased eosinophils in adipose tissue of metabolic syndrome. J Diabetes Complications (2019) 33:535–8. 10.1016/j.jdiacomp.2019.05.010 31204245

[51] Goh YPS Henderson NC Heredia JE Red Eagle A Odegaard JI Lehwald N Eosinophils secrete IL-4 to facilitate liver regeneration. Proc Natl Acad Sci U S A (2013) 110:9914–9. 10.1073/pnas.1304046110 PMC3683773 23716700

[52] Heredia JE Mukundan L Chen FM Mueller AA Deo RC Locksley RM Type 2 innate signals stimulate fibro/adipogenic progenitors to facilitate muscle regeneration. Cell (2013) 153:376–88. 10.1016/j.cell.2013.02.053 PMC3663598 23582327

[53] Pereira-Dutra FS Teixeira L de Souza Costa MF Bozza PT Fat, fight, and beyond: The multiple roles of lipid droplets in infections and inflammation. J Leukoc Biol (2019) 106:563–80. 10.1002/JLB.4MR0119-035R 31121077

[54] Bozza PT Bakker-Abreu I Navarro-Xavier RA Bandeira-Melo C Lipid body function in eicosanoid synthesis: an update. Prostagland Leukot Essent Fatty Acids (2011) 85:205–13. 10.1016/j.plefa.2011.04.020 21565480

[55] Melo RCN Weller PF Lipid droplets in leukocytes: Organelles linked to inflammatory responses. Exp Cell Res (2016) 340:193–7. 10.1016/j.yexcr.2015.10.028 PMC4744558 26515551

[56] Lund SJ Portillo A Cavagnero K Baum RE Naji LH Badrani JH Leukotriene C4 Potentiates IL-33-Induced Group 2 Innate Lymphoid Cell Activation and Lung Inflammation. J Immunol Baltim Md 1950 (2017) 199:1096–104. 10.4049/jimmunol.1601569 PMC5531601 28667163

[57] Bandeira-Melo C Hall JC Penrose JF Weller PF Cysteinyl leukotrienes induce IL-4 release from cord blood-derived human eosinophils. J Allergy Clin Immunol (2002) 109:975–9. 10.1067/mai.2002.124269 12063527

[58] Reginato MJ Krakow SL Bailey ST Lazar MA Prostaglandins promote and block adipogenesis through opposing effects on peroxisome proliferator-activated receptor gamma. J Biol Chem (1998) 273:1855–8. 10.1074/jbc.273.4.1855 9442016

[59] Rahman MS Prostacyclin: A major prostaglandin in the regulation of adipose tissue development. J Cell Physiol (2019) 234:3254–62. 10.1002/jcp.26932 30431153

[60] Elieh Ali Komi D Shafaghat F Christian M Crosstalk Between Mast Cells and Adipocytes in Physiologic and Pathologic Conditions. Clin Rev Allergy Immunol (2020) 58:388–400. 10.1007/s12016-020-08785-7 32215785 PMC7244609

[61] Milling S Adipokines and the control of mast cell functions: from obesity to inflammation? Immunology (2019) 158:1–2. 10.1111/imm.13104 31429086 PMC6700462

[62] Żelechowska P Wiktorska M Różalska S Stasikowska-Kanicka O Wągrowska-Danilewicz M Agier J Leptin receptor is expressed by tissue mast cells. Immunol Res (2018) 66:557–66. 10.1007/s12026-018-9029-0 PMC6244893 30269202

[63] Żelechowska P Agier J Różalska S Wiktorska M Brzezińska-Błaszczyk E Leptin stimulates tissue rat mast cell pro-inflammatory activity and migratory response. Inflammation Res Off J Eur Histamine Res Soc Al (2018) 67:789–99. 10.1007/s00011-018-1171-6 PMC6096628 30019195

[64] Xue L Gyles SL Wettey FR Gazi L Townsend E Hunter MG Prostaglandin D2 causes preferential induction of proinflammatory Th2 cytokine production through an action on chemoattractant receptor-like molecule expressed on Th2 cells. J Immunol Baltim Md 1950 (2005) 175:6531–6. 10.4049/jimmunol.175.10.6531 16272307

[65] Palhinha L Liechocki S Hottz ED Pereira JA da S de Almeida CJ Moraes-Vieira PMM Leptin Induces Proadipogenic and Proinflammatory Signaling in Adipocytes. Front Endocrinol (2019) 10:841. 10.3389/fendo.2019.00841 PMC6923660 31920961

[66] Peeraully MR Sievert H Bulló M Wang B Trayhurn P Prostaglandin D2 and J2-series (PGJ2, Delta12-PGJ2) prostaglandins stimulate IL-6 and MCP-1, but inhibit leptin, expression and secretion by 3T3-L1 adipocytes. Pflugers Arch (2006) 453:177–87. 10.1007/s00424-006-0118-x 16924534

[67] Xue L Salimi M Panse I Mjösberg JM McKenzie ANJ Spits H Prostaglandin D2 activates group 2 innate lymphoid cells through chemoattractant receptor-homologous molecule expressed on TH2 cells. J Allergy Clin Immunol (2014) 133:1184–94. 10.1016/j.jaci.2013.10.056 PMC3979107 24388011

[68] Mjösberg JM Trifari S Crellin NK Peters CP van Drunen CM Piet B Human IL-25- and IL-33-responsive type 2 innate lymphoid cells are defined by expression of CRTH2 and CD161. Nat Immunol (2011) 12:1055–62. 10.1038/ni.2104 21909091

[69] Virtue S Masoodi M de Weijer B a M van Eijk M Mok CYL Eiden M Prostaglandin profiling reveals a role for haematopoietic prostaglandin D synthase in adipose tissue macrophage polarisation in mice and humans. Int J Obes 2005 (2015) 39:1151–60. 10.1038/ijo.2015.34 PMC4486370 25801691

