# Supplementary figures and images for: Leptin Elicits In Vivo Eosinophil Migration and Activation: Key Role of Mast Cell-Derived PGD2
Source: Front Endocrinol (Lausanne). 2020 Sep 29;11:572113. doi: 10.3389/fendo.2020.572113 (PMC7551309; doi:10.3389/fendo.2020.572113)

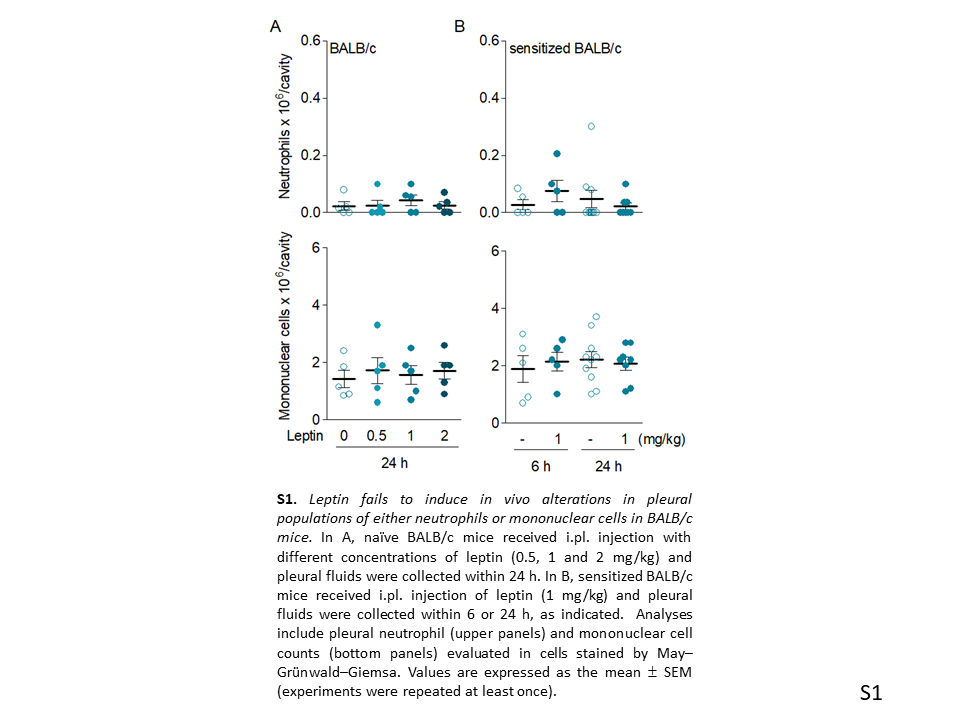

Supplement: Supplementary file 1 [file Image_1.tif]

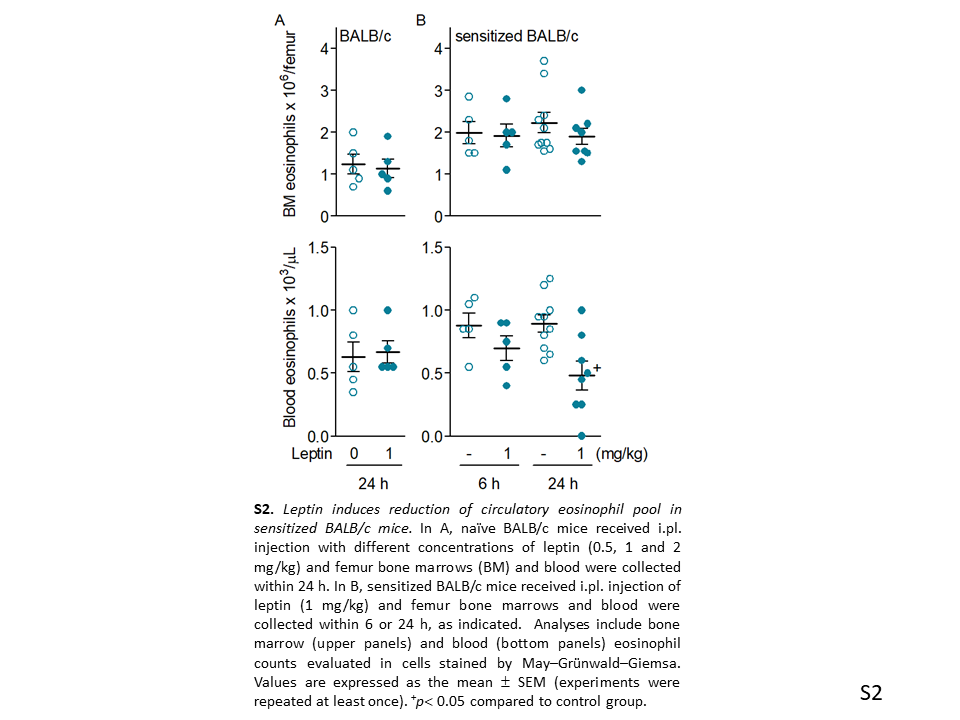

Supplement: Supplementary file 2 [file Image_2.tif]

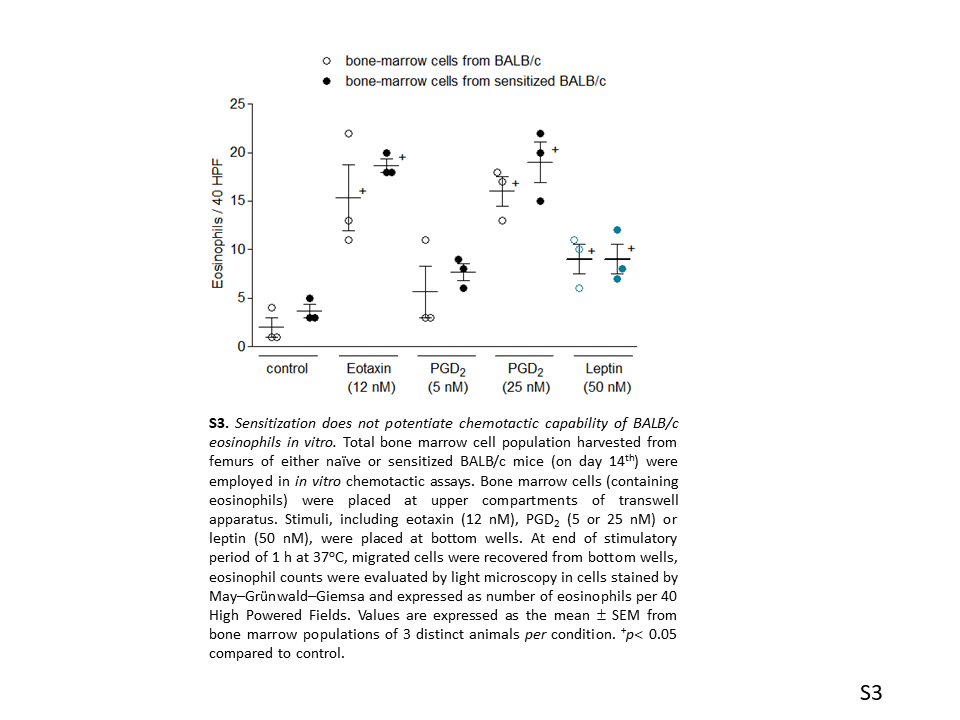

Supplement: Supplementary file 3 [file Image_3.tif]
